# Transcriptomic and genomic characteristics of intrahepatic metastases of primary liver cancer

**DOI:** 10.1186/s12885-024-12428-x

**Published:** 2024-06-01

**Authors:** Weilong Zou, Zhanjie Fang, Yu Feng, Shangjin Gong, Ziqiang Li, Meng Li, Yong Sun, Xiuyan Ruan, Xiangdong Fang, Hongzhu Qu, Haiyang Li

**Affiliations:** 1 Department of Hepatobiliary Surgery, The Affiliated Hospital of Guizhou Medical University, Guiyang, Guizhou China; 2 CAS Key Laboratory of Genome Sciences and Information, Beijing Institute of Genomics, Chinese Academy of Sciences/China National Center for Bioinformation, Beijing, China; 3 University of Chinese Academy of Sciences, Beijing, China

**Keywords:** Hepatocellular carcinoma (HCC), Metastatic, CIBERSORTx, Tumor microenvironment, Portal vein thrombus

## Abstract

**Background:**

Patients with primary multifocal hepatocellular carcinoma (HCC) have a poor prognosis and often experience a high rate of treatment failure. Multifocal HCC is mainly caused by intrahepatic metastasis (IM), and though portal vein tumor thrombosis (PVTT) is considered a hallmark of IM, the molecular mechanism by which primary HCC cells invade the portal veins remains unclear. Therefore, it is necessary to recognize the early signs of metastasis of HCC to arrange better treatment for patients.

**Results:**

To determine the differential molecular features between primary HCC with and without phenotype of metastasis, we used the CIBERSORTx software to deconvolute cell types from bulk RNA-Seq based on a single-cell transcriptomic dataset. According to the relative abundance of tumorigenic and metastatic hepatoma cells, *VEGFA*^*+*^ macrophages, effector memory T cells, and natural killer cells, HCC samples were divided into five groups: Pro-T, Mix, Pro-Meta, NKC, and MemT, and the transcriptomic and genomic features of the first three groups were analyzed. We found that the Pro-T group appeared to retain native hepatic metabolic activity, whereas the Pro-Meta group underwent dedifferentiation. Genes highly expressed in the group Pro-Meta often signify a worse outcome.

**Conclusions:**

The HCC cohort can be well-typed and prognosis predicted according to tumor microenvironment components. Primary hepatocellular carcinoma may have obtained corresponding molecular features before metastasis occurred.

**Supplementary Information:**

The online version contains supplementary material available at 10.1186/s12885-024-12428-x.

## Background

HCC is the sixth most common malignant tumor and the third leading cause of mortality worldwide [1]. In recent decades, approximately 0.25–1 million patients have been diagnosed with HCC annually. Moreover, 41–75% of HCC cases are initially diagnosed as multifocal tumors with limited interventions, indicating difficult clinical management and poor diagnosis [2]. Multifocal HCCs are classified into multicenter (MC) or intrahepatic metastatic (IM) tumors [3, 4]. The former is often found in the early stages, while the latter refers to tumors that have already metastatically spread before diagnosis [2]. Significant differences in biological behaviors, treatment selection, and prognosis between IM and MC tumors demonstrate the clinical importance of classification [3, 4]. For IM tumors, portal vein tumor thrombus (PVTT) is considered an undeniable factor associated with the terminal stage of HCCs [5]. Several studies have focused on the differences in molecular characteristics, such as gene mutations [6], gene expression [7–9], long non-coding RNAs (lncRNAs) [10], and epigenetic modifications [11], between PVTT and primary tumors (PTs). Ye et al. found no significant difference between the expression profiles of PTs and matched PVTTs [7], whereas another study reached different conclusions on lncRNAs [10]. To avoid the impact of pronounced heterogeneity among patients, an individualized differential analysis revealed 20 genes that co-vary in multiple patients and potentially contribute to HCC invasion [11].

These results indicate that hepatoma cells in situ may undergo molecular changes before invading blood vessels. Based on this hypothesis, using a single-cell RNA sequencing (scRNA-Seq) dataset of HCC with or without PVTTs, we predicted the cellular composition of a large bulk RNA-Seq HCC cohort and classified all samples into five subgroups: Pro-T, Mix, Pro-Meta, NKC, and MemT. The Pro-T group was enriched in tumorigenic hepatocytes, while the Pro-Meta group was enriched in metastatic hepatocytes. In the Mix group, there was no significant difference in the abundance of tumorigenic and metastatic hepatocytes. The NKC and MemT groups are defined by high levels of natural killer cells and effector memory T cells, respectively. According to survival analysis, primary HCC with metastatic features had the worst prognosis. Many differential mutations and genes were identified between the Pro-T and Pro-Meta groups.

## Results

### An immunosuppressive and metastasis-prone HCC microenvironment contributes to a poor prognosis

**Fig. 1:**
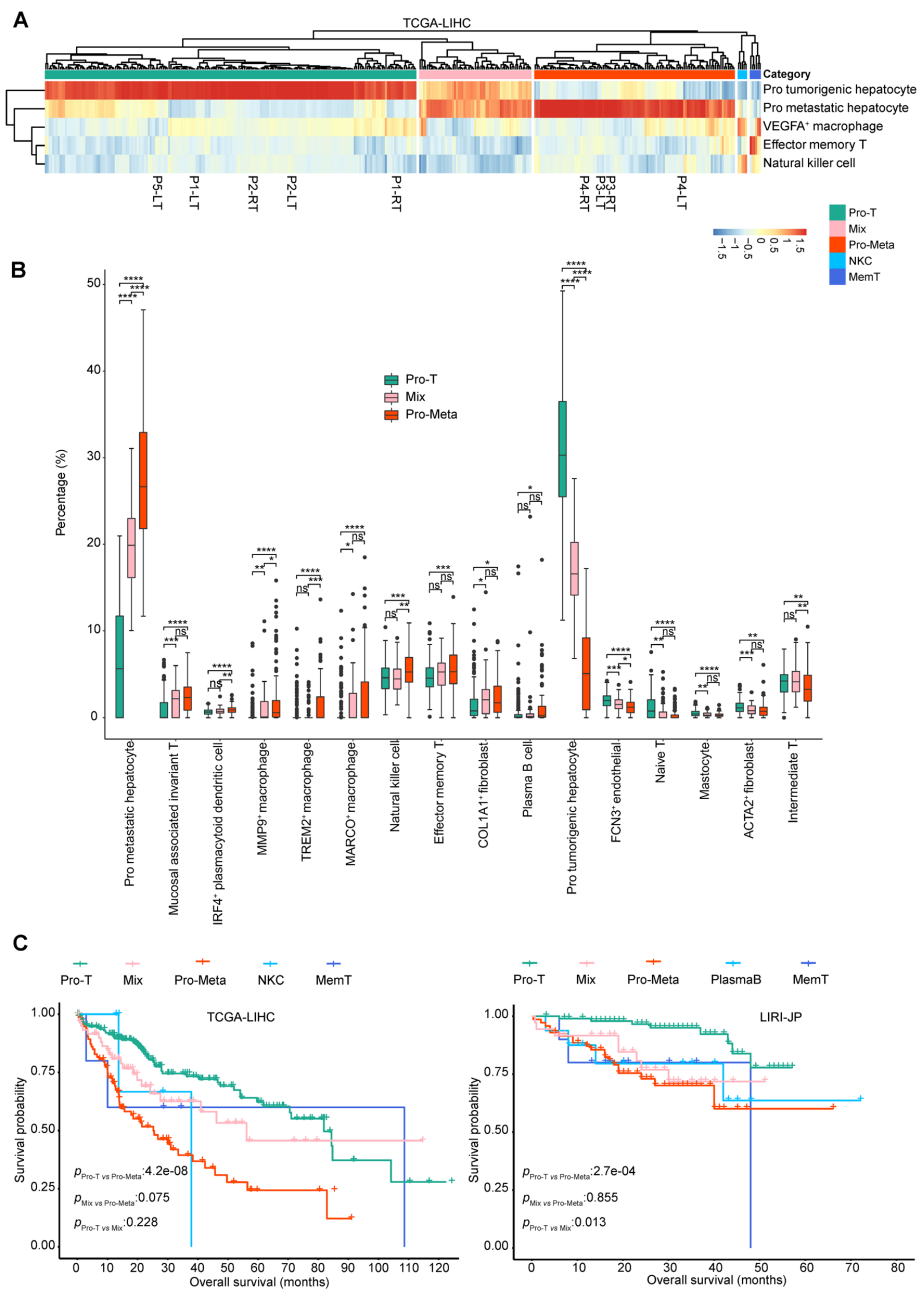
Stratification of patients with hepatocellular carcinoma (HCC). **A** Hierarchical clustering of HCC samples based on top five relative cell abundances. The native samples are highlighted. **B** Relative cell abundance changes in three HCC groups. ns: *p* > 0.05, *: *p* ≤ 0.05, **: *p* ≤ 0.01, ***: *p* ≤ 0.001, ****: *p* ≤ 0.0001; Student’s t-test. **C** Survival analyses based on five HCC groups in cohort TCGA-LIHC and LIRI-JP; Log-Rank test

We performed bulk RNA-Seq on five native patients with liver cancer, including nine PT samples. Two of the patients (P3 and P4) were diagnosed with HCC combined with PVTT (Additional file 1: Table S1). To enhance statistical power, we integrated RNA-Seq data from both our and The Cancer Genome Atlas (TCGA) liver hepatocellular carcinoma (LIHC) cohorts (primary cancer samples only, *n* = 374) using the R package sva, as described in the Methods. Bulk RNA-Seq data were deconvoluted using single-cell gene expression signatures to characterize the microenvironment of the HCC cohort. It’s hard to ascertain whether the patients in the TCGA-LIHC cohort were diagnosed with PVTT. Therefore, to divide the TCGA-LIHC samples into meaningful groups, we selected independent scRNA-Seq datasets with similar clinical characteristics to the native patients, that is, the samples were taken from patients with and without PVTT. As a supplement, we also included the International Cancer Genome Consortium liver cancer-RIKEN, Japan (ICGC LIRI-JP, *n* = 240) and France (ICGC LICA-FR, *n* = 159) cohorts in the analysis. Based on the relative TME cell scores of the top five cell types, patients in cohort TCGA-LIHC were divided into five groups, i.e., Pro-T, Mix, Pro-Meta, NKC, and MemT, using unsupervised hierarchical clustering (Fig. 1A), and the number of patients in each group was 202 (52.7%), 61 (15.9%), 109 (28.5%), 5 (1.3%), and 6 (1.6%), respectively. P1, P2, and P5 were classified as group Pro-T while P3 and P4 were classified as group Pro-Meta, which is consistent with the clinical truth (Additional file 1: Table S1). The Pro-T group showed high tumorigenic hepatocyte infiltration, whereas the Pro-Meta group had high levels of metastatic hepatocytes. The Mix group exhibited both of these characteristics. The remaining two groups were named MemT and NKC owing to the high abundance of effector memory T cells and natural killer cells, respectively. We observed a similar clustering result in cohort LIRI-JP, where the samples were divided into five groups, too, including Pro-T (*n* = 106, 42.6%), Mix (*n* = 39, 15.7%), Pro-Meta (*n* = 78, 31.3%), PlasmaB (*n* = 16, 6.4%), and MemT (*n* = 10, 4.0%) (Additional file 2: Fig. S1A, upper). In cohort LICA-FR, 101 (60.1%) samples were classified as group Pro-T, 52 (31.0%, group Pro-Meta), and 10 (6.0%, group Pro-Meta2) samples were considered related to metastatic characteristics, and only 3 and 2 samples were classified as group VEGFA^+^ Macro and MemT, respectively (Additional file 2: Fig. S1A, lower).

By using either the top five or all cell type component fractions as inputs for principal component analysis (PCA), we observed that the samples were separated from each other clearly in three cohorts (Additional file 2: Fig. S1B, left and middle plots). To further confirm the rationality of our classification methods, we performed PCA based on gene expression features. In cohort TCGA-LIHC and LIRI-JP, we observed that samples from the Pro-T and Pro-Meta groups were more likely to gather together within the group and separate between the groups, and the samples from the Mix group were scattered between the Pro-T and Pro-Meta groups, suggesting a transitional stage of Pro-Meta. However, in cohort LICA-FR, it is difficult to identify samples in transition state as Mix group, which may be attributed to its smaller sample size (Additional file 2: Fig S1B, right).

We focused on the differences in cell components between the Pro-T and Pro-Meta groups in cohort TCGA-LIHC because of the largest number of samples and the greatest difference between them (Fig. 1B). The number of mucosal-associated invariant T (MAIT) cells and natural killer cells increased from Pro-T to Pro-Meta, suggesting that innate immunity may play a role in constructing the TME of the Pro-Meta group. Duan et al. found that tumor-educated MAIT cells can promote tumors by upregulating inhibitory factors, such as CTLA-4 [12]. Three types of macrophages, *MMP9*^*+*^, *TREM2*^*+*^, and *MARCO*^*+*^, were more abundant in the Pro-Meta group (Fig. 1B). Several studies have shown that *TREM2*^*+*^ macrophages promote immune suppression [13] and play a role in transforming the TME into an anti-inflammatory state, leading to the growth of HCC [14, 15]. Lu et al. showed that *MMP9*^*+*^ macrophages are in the late stage of macrophage differentiation and promote HCC progression by inducing tumor cell migration and angiogenesis [9].

Compared with the Pro-T group, the fractions of effector memory T and plasma B cells were significantly increased in the Pro-Meta group, suggesting a shift towards the terminal phase of the immune response. In contrast, the proportion of naïve T cells decreased from Pro-T to Pro-Meta. *FCN3*^*+*^ endothelial cells and *ACTA2*^*+*^ fibroblasts are significantly lost when the phenotype becomes metastatic, indicating that the blood vessels may be broken and the formation of tumor thrombus is promoted. We further investigated the changes in cellular composition between the Pro-T and Pro-Meta groups across different cohorts. Except for tumorigenic and metastatic hepatocytes, only the component of MMP9^+^ macrophage showed increasing in the Pro-Meta group across three cohorts consistently, indicating the heterogeneity of tumor microenvironment composition among patients (Additional file 3: Fig S2A).

Furthermore, we performed a survival analysis to compare the prognosis of patients in the different groups. In cohort TCGA-LIHC, the median survival time was 81.9, 25.2, and 56.2 months in the Pro-T, Pro-Meta, and Mix groups, respectively, indicating the worst prognosis in the Pro-Meta group and the transitional stage of the Mix group (Fig. 1C, left). We observed a similar trend in cohort LIRI-JP, too (Fig. 1C, right). Overall, the comparative analysis of the HCC TME components indicated a suppressive and anti-inflammatory immune signature in the Pro-Meta group. The absence of some stromal cells may promote the detachment of HCC cells from the primary focus.

### Gene co-expression modules associated with the Pro-Meta and Pro-T phenotype

**Fig. 2:**
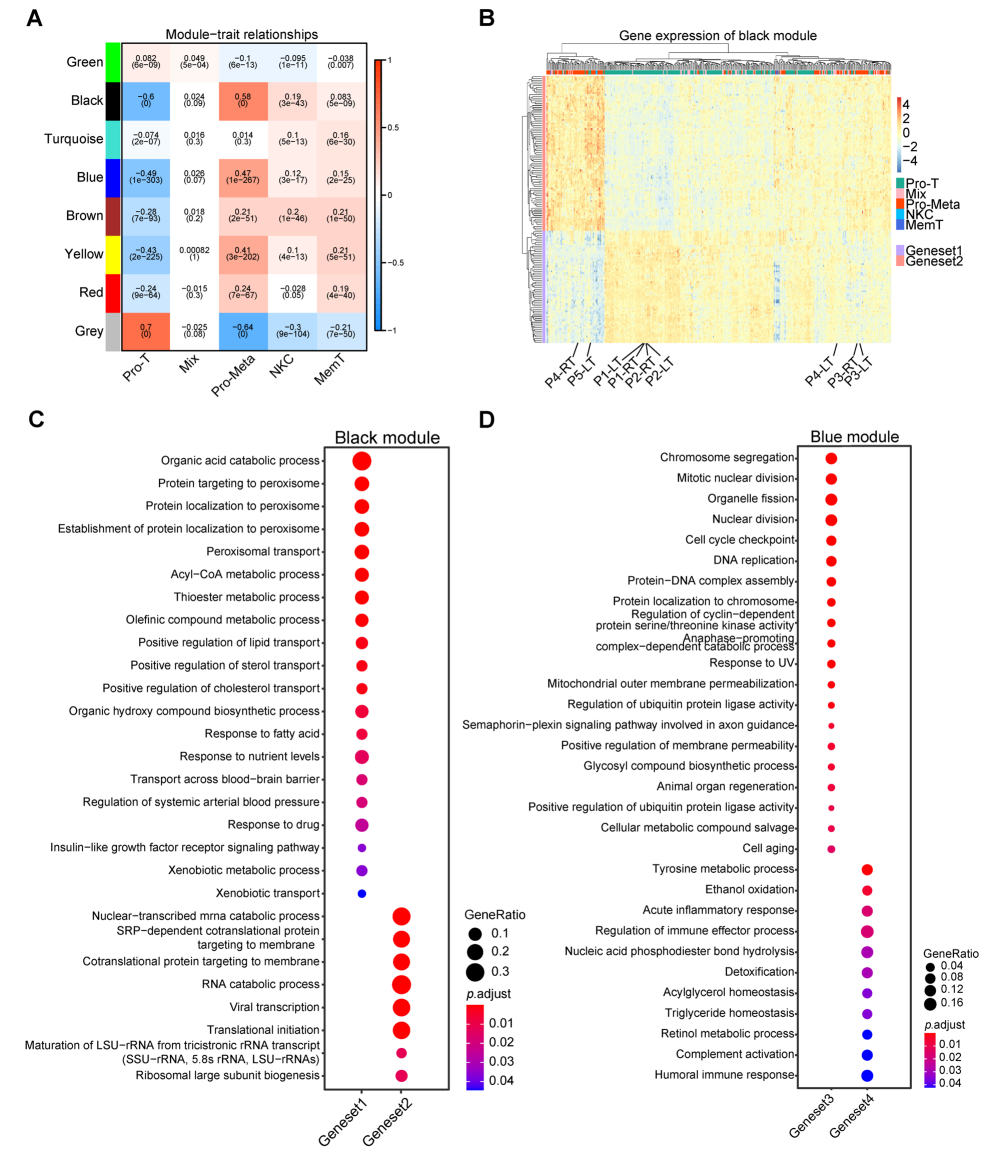
Weighted correlation network analysis (WGCNA) of the hepatocellular carcinoma (HCC) cohort. **A** Correlation heatmap of gene modules and group information in the HCC cohort. **B** The relative expression of genes in the black module. Genes are clustered into two groups, geneset1 and geneset2, based on their expression levels. The native samples are highlighted. **C, D** Gene ontology (GO) analysis of genesets in the black (**C**) or blue (**D**) module

To further determine the relationship between gene expression features and HCC phenotypes, we performed the weighted gene correlation network analysis (WGCNA). After average linkage hierarchical clustering, eight co-expression modules were identified, among them, genes in the grey module cannot be clustered in any other module, thus representing no consistent biological functions. The black, blue, and yellow modules show significant positive correlations (*p* < 0.05, cor > 0.3) with the Pro-Meta phenotype, whereas these modules exhibit significant negative correlations (*p* < 0.05, cor < -0.3) with the Pro-T phenotype (Fig. 2A). For phenotype Pro-Meta and Pro-T, the high correlation between module membership in black and blue modules and the gene significance imply the essential role of these gene modules. However, the correlations in the yellow module are low (Pro-Meta: cor = -0.24; Pro-T: cor = -0.18), thus it is abandoned (Additional file 4: Fig. S3A, B).

Due to the “unsigned” network type we selected, we further measured the relative expression of genes in two modules between groups Pro-T and Pro-Meta. Genes were subsequently divided into four sets, of which genesets 1 and 4 were highly expressed in the Pro-T group, whereas genesets 2 and 3 were highly expressed in the Pro-Meta group (Fig. 2B and Additional file 4: Fig. S3C). Genesets 1 and 4 were involved in normal liver functions such as organic acid catabolic processes, peroxisomal transport, lipid metabolism, metabolism of small and macromolecular compounds, and amino acid metabolism. We also noticed inflammation and humoral immune response pathways, which may be due to patients with HCC usually present with hepatitis. Genesets 2 and 3 were associated with cell replication, division, and disruptions in cellular metabolism, which are characteristics commonly observed in more malignant HCC cells (Fig. 2C and D). Collectively, our results suggested that the Pro-Meta group exhibits a more malignant phenotype, manifested by the loss of hepatocyte metabolism and the acquisition of cell proliferation traits.

### Different functions of the Pro-T and Pro-Meta groups

**Fig. 3:**
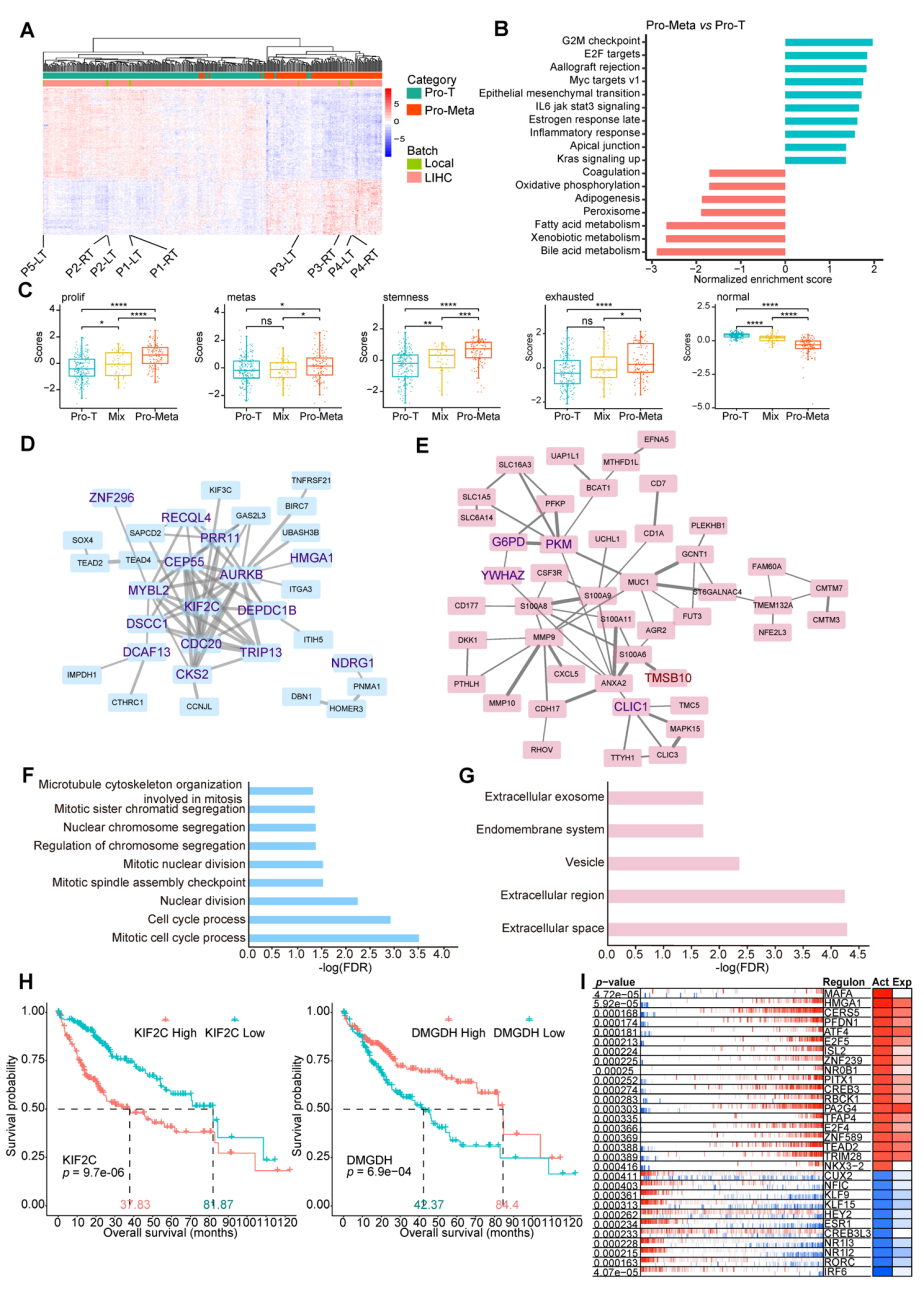
Differential gene expression analysis of hepatocellular carcinoma (HCC) groups. **A** Gene expression heatmap of differentially expressed genes (DEGs) between the Pro-T and Pro-Meta groups. The native samples are highlighted. **B** GSEA of genes from the Pro-T and Pro-Meta groups. The functional terms enriched in the Pro-Meta group with enrichment scores greater than 0 were shown in cyan bars, and those in the Pro-T group with enrichment scores less than 0 were shown in red bars. **C** ssGSEA of the proliferation, metastasis, stemness, exhaustion, and normal scores in samples from the Pro-T, Mix, and Pro-Meta groups. **D, E** STRING database clustering analysis of genes upregulated in the Pro-Meta group resulting in two distinct biological meaningful clusters: cluster M1 (**D**) and cluster M2 (**E**). Genes in the PPI network that intersect with the black and blue gene modules from WGCNA that are upregulated in the Pro-Meta group (genesets 2 and 3) are shown in maroon and indigo, respectively. **F, G** GO analysis of cluster M1 (**F**) and cluster M2 (**G**), respectively. **H** Survival analysis based on *KIF2C* and *DMGDH* expressions. High (red) and low (cyan) expression groups were separated by the median of gene expression levels. The median survival for each group is highlighted. **I** Differential activity analysis of transcription factor (TF) proteins between the Pro-T and Pro-Meta groups. Red and blue bars represent the activated and suppressed target genes of the transcription factor (TF), respectively. The Act and Exp color blocks represent the relative TF protein activity and TF gene expression, respectively. The TFs with high activity or expression in the Pro-Meta group are marked in red, whereas those with high activity or expression in the Pro-T group are marked in blue. The activity or expression level of TFs is positively correlated with the color depth

Next, we compared the differences in gene expression between patients from the Pro-T and Pro-Meta groups. Among DEGs (adjusted *p* < 5e-2 and |log2FC(Pro-Meta/Pro-T)| > 1) found in all cohorts, a considerable number of them coexist in at least two cohorts (Additional file 3: Fig. S2B). Importantly, among 368 co-upregulated and 281 co-downregulated DEGs, the trend of logarithmic fold change of them is consistent (Additional file 3: Fig. S2C). To obtain clearer results, we conducted the subsequent analysis based on cohort TCGA-LIHC with a more stringent threshold and identified 510 DEGs (adjusted *p* < 1e-25 and |log_2_FC(Pro-Meta/Pro-T)| > 1). The two groups clustered well according to DEGs, and the batch effect was removed (Fig. 3A). Consistent with the WGCNA results, pathways related to the cell cycle, cell proliferation, and growth, such as the G2M checkpoint, E2F targets, and Myc targets v1, were highly activated in the Pro-Meta group. As expected, the epithelial-mesenchymal transition pathway was also enriched in the Pro-Meta group, which supported its metastatic character. In contrast, metabolic and synthetic processes of multiple substances were upregulated in the Pro-T group (Fig. 3B).

Single-sample gene set enrichment analysis (ssGSEA) showed that the scores for proliferation, metastasis, stemness, and immune exhaustion of HCC cells constantly increased from the Pro-T group to the Pro-Meta group. When we used genes that were specifically expressed in normal human liver tissue to calculate the “normal liver function” scores of different groups, we found that the scores decreased from the Pro-T group to the Pro-Meta group, suggesting that the hepatoma cells dedifferentiate during tumor metastasis (Fig. 3C). Thus, our results showed that the acquisition of a metastatic phenotype by primary HCC cells was a progressive process.

To explore whether the DEGs reflected the biological significance of the tumor, we performed a protein-protein interaction (PPI) analysis. By using the k-means method, we found two meaningful clusters in the PPI network of group Pro-Meta, M1 and M2, one representing high-level cell cycle pathway activity at the biological process level (Fig. 3D and F, cluster M1), the other representing the establishment of the extracellular matrix and the cell membrane at the cellular component level (Fig. 3E and G, cluster M2). The genes involved in the Pro-Meta PPI network were intersected with genes in the black and blue modules, and the upregulated genes obtained from WGCNA (genesets 2 and 3) were also upregulated in the network, whereas genes in genesets 1 and 4 didn’t appear in Pro-Meta network at all. Specifically, 19 genes were upregulated in both the PPI network and the blue module of the Pro-Meta group, 15 of which belonged to cluster M1 and 4 belonged to cluster M2. It is worth mentioning that *TMSB10* (Thymosin Beta-10), which plays an important role in the organization of the cytoskeleton, was upregulated in both cluster M2 and the black module of the Pro-Meta group.

In the Pro-T group, we found six meaningful clusters, T1 to T6, most of which were related to metabolic processes according to the GO enrichment results (Additional file 5: Fig. S4A-C and Additional file 6: Fig. S5A-B, cluster T1 to T5). Genes in the Pro-T network were also intersected with genes in the WGCNA black and blue modules, interestingly, the co-upregulated genes all came from genesets 1 and 4, and were colored dark olive green and dark turquoise, respectively. The above results indicate that there are significant differences in gene expression between phenotype Pro-Meta and Pro-T. The genes in cluster T6 appeared to be closely associated with fibrinolysis, negative regulation of blood coagulation, and wound healing (Additional file 6: Fig. S5C, cluster T6). Coagulation activation is closely associated with the malignant phenotype of tumors [16]; Anticoagulants can inhibit angiogenesis and the growth of many cancers [17]. More importantly, thrombomodulin, a natural anticoagulant, may inhibit the formation of PVTTs and the occurrence of IM [18]. The formation of portal vein thrombosis is infrequent in intrahepatic cholangiocarcinoma and distant metastatic liver cancer [19]. Li et al. suggested that IM may be a unique feature of HCC caused by an imbalance of the coagulation and anticoagulation systems in liver cells [20]. Taken together, these results suggest that the loss of anticoagulant ability of the TME in the Pro-Meta group promoted the formation of portal vein tumor thrombi.

In general, 75 genes coexisted in the PPI network clusters and either the black or blue gene modules from WGCNA. We evaluated the potential of the 75 intersecting genes as prognostic factors and divided the HCC samples into two groups based on the median expression of each gene. After performing survival analysis, we found that all genes that were upregulated in the Pro-Meta group and co-occurred in the PPI and WGCNA networks were predictive of lower median survival times, whereas all genes upregulated in the Pro-T group were predictive of higher median survival times. Among the genes that exhibited significant differences (log-rank test, *p* < 0.05) between the survival curves, we identified 25 and 24 genes that could serve as significant high- and low-risk prognostic factors, respectively, in the HCC cohorts. As expected, samples from the better and poorer prognosis groups typically infiltrated with lower and higher levels of metastatic hepatocytes, respectively. The proportion of metastatic hepatocytes in the TME of the good prognosis group ranged from 2.7 to 16.7% with a median of 9.7% and sd of 0.03. In contrast, the proportion of metastatic hepatocytes in the TME of the poor prognosis group ranged from 39.2 to 53.2% with a median of 46.2% and sd of 0.03 (Additional file 7: Table S2). According to their *p* values, high *KIF2C* expression implied the worst outcome, whereas high expression of dimethylglycine dehydrogenase (*DMGDH)* implied the best outcome (Fig. 3H). *KIF2C* (Kinesin Family Member 2 C) is an important regulator of the cell cycle; and is highly expressed in a variety of tumors, such as Endometrial cancer [21], gliomas [22], breast cancer [23], and lung cancer [24]; and has been shown to participate in tumor progression and metastasis. In vitro experiments have shown that *KIF2C* activates the *MEK/ERK* pathway to promote the invasion of HCC [25] through epithelial-mesenchymal transition [26]. *DMGDH* inhibits metastasis and induces apoptosis in HCC cells [27, 28].

To further determine the differences in transcriptional regulation between the Pro-T and Pro-Meta groups, we performed an enriched regulon analysis. The transcription factors *E2F5* and *E2F4*, which are associated with the cell cycle and proliferation, were upregulated in the Pro-Meta group. In addition, we noticed that the second-place regulon *HMGA1* (High Mobility Group AT-Hook 1) was also upregulated in the PPI network and blue module of the Pro-Meta group. Many studies have reported that *HMGA1* is involved in the metastatic progression of HCC cells [29–32] (Fig. 3I). However, regulons that were upregulated in the Pro-T group, such as *CREB3L3*, *NR1I3*, and *NR1I2*, are more likely to participate in the metabolism of endogenous and exogenous compounds. These findings are consistent with the gene set enrichment analysis (GSEA) and PPI data.

### Gene mutation characteristics of metastatic HCC

**Fig. 4:**
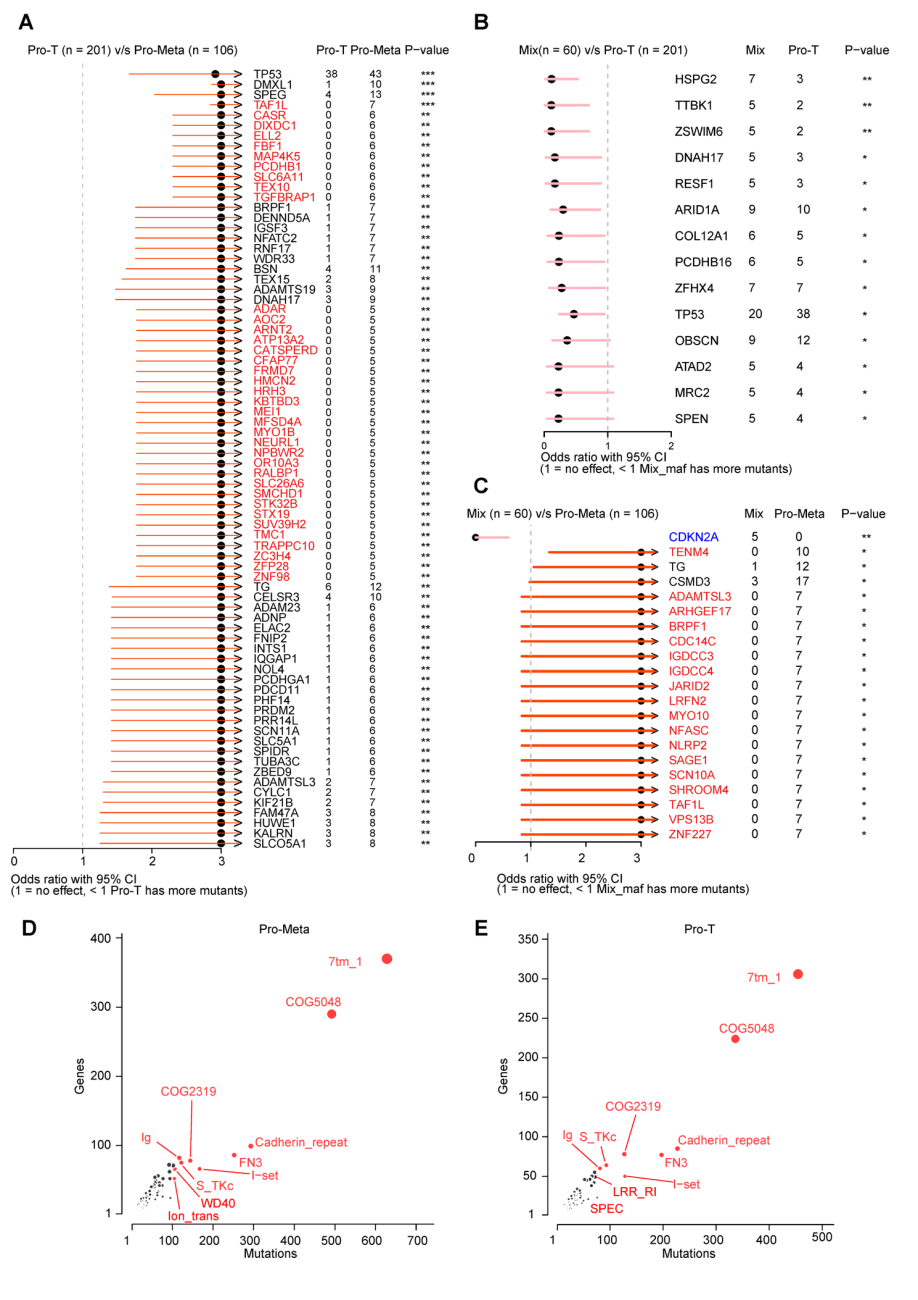
Differentially mutated genes and domains between the hepatocellular carcinoma (HCC) groups. **A** Differentially mutated genes between the Pro-T and Pro-Meta groups. The bars represent the 95% confidence interval of the odds ratio, and the adjacent table displays the number of samples in the Pro-T and Pro-Meta groups that contain mutations in the highlighted gene. Genes specifically mutated in the Pro-Meta group are colored in red. **: *p* ≤ 0.01, ***: *p* ≤ 0.001; Fisher’s exact test. **B** Differentially mutated genes between the Mix and Pro-T groups. **C** Differentially mutated genes between the Mix and Pro-Meta groups. Genes specifically mutated in the Mix and pro-Meta groups are colored in blue and red, respectively. **D, E** Frequently mutated pfam protein domains in the Pro-Meta and Pro-T groups, respectively. The Y-axis represents the number of genes containing a specified domain. The X-axis represents the number of mutations that occur in the domain. The top ten domains are highlighted in red. The size of each bubble is proportional to the number of genes that contain the highlighted domain

We calculated the number of mutations that occurred in each gene within three groups (Pro-T, Mix, and Pro-Meta) stratified from native and TCGA-LIHC samples and compared the mutation load differences of each gene using a pairwise comparison. A total of 199 differentially mutated genes were identified after comparing groups Pro-T and Pro-Meta (*p* < 0.05, Fisher’s exact test). Notably, all differentially mutated genes between Pro-T and Pro-Meta presented higher mutation frequencies in the Pro-Meta group, and 37 genes (48.7%) were specifically mutated in the Pro-Meta group, suggesting the possibility of a new phenotypic acquisition in this group. TP53 mutations are frequent in HBV-infected liver cancer [33], and a higher frequency of these mutations in the Pro-Meta group implies a more malignant phenotype (Fig. 4A). However, when mutations were compared between the Mix and Pro-T groups, differentially mutated genes were found specifically in the Mix group (Fig. 4B). When it comes to group Mix vs. Pro-Meta, *CDKN2A* (Cyclin Dependent Kinase Inhibitor 2 A) seems specifically mutated in the Mix group and is known to be an important tumor suppressor gene, indicating a disordered cell cycle in this group (Fig. 4C).

Domain enrichment analysis showed that the top ten mutated protein domains were similar between the Pro-T and Pro-Meta groups. Notably, the PF00520 domain (Ion_trans) was more frequently affected in Pro-Meta, with 105 mutations across 52 genes (Fig. 4D), compared to Pro-T, which had 69 mutations across 42 genes (Fig. 4E). The Ion_trans domain belongs to a family of six transmembrane helicases responsible for ion transportation and is primarily involved in ion signaling (Na^+^/Ca^2+^) and the interaction pathways of *L1CAM*. Another differentially mutated protein domain was WD40, with 105 mutations across 66 genes (Fig. 4D) in the Pro-Meta group and 71 mutations across 55 genes in the Pro-T group (Fig. 4E). The WD40 domain is found in several eukaryotic proteins that perform a wide variety of functions, including serving as an adaptor/regulatory module in signal transduction, participating in pre-mRNA processing, and aiding in cytoskeleton assembly. This may explain the lack of metabolic ability for multiple substances in liver cancer cells of the Pro-Meta group at the genomic level.

## Discussion

We conducted a thorough investigation of the medical records of our patients and found that both P3 and P4 had portal vein tumor thrombi during surgery. Because of the small difference between the PT and PVTTs in HCC reported in previous studies, we assumed that PTs that have shown invasion of the portal vein are different from those that remain silent.

With the rapid progress in single-cell RNA sequencing, tumor heterogeneity can be studied at the single-cell level. Thus, to test our hypothesis, we analyzed the TME for bulk RNA-Seq samples using a scRNA-Seq dataset with PT and PVTT samples. To improve statistical power, we included the TCGA HCC cohort in the analysis. Downstream analysis was based on groups classified according to the relative abundance of cells from the bulk RNA-Seq datasets.

Both cellular components and expression profile showed that samples P3 and P4 were gathered together properly and enriched with metastatic liver cancer cells. Downstream analysis was based on groups classified according to the relative abundance of cells. We also found that the number of several types of stromal cells increased or decreased with changes in the relative abundance of liver cancer cells changes, indicating a more immunosuppressive and invasive TME of samples with a metastatic phenotype. The module-trait correlation analysis showed that compared with the silent PT samples, the samples with a metastatic phenotype were more proliferative and had lower liver function. In contrast to previous studies, we found a batch of differentially expressed genes upregulated in the Pro-Meta group, which represents the highly proliferative and activated extracellular matrix organization pathway. The GSEA and ssGSEA results also implied overall dedifferentiation in the Pro-Meta group.

There are several limits of this study: First, the stratification of patients is largely determined by the accuracy of the deconvolution algorithm. In this study, we adopted the most widely used algorithm and used strict quality control measures, such as performing batch correction and setting larger permutations for significance analysis to mitigate the potential biases in the inference of cellular composition. Nonetheless, the absolute accuracy of CIBERSORTx can not be guaranteed. Second, more detailed clinical information on patients is needed to judge the accuracy of unsupervised clustering, however, due to the lack of bulk and single-cell RNA-Seq datasets about unpaired PT and PVTT samples, we have not been able to perform further validation. Besides, due to the missing etiological investigation about patients in three cohorts, we focused more on the occurrence of PVTT, and placed less emphasis on the etiology of HCC, i.e., whether it was caused by HBV, HCV, or other factors. Finally, it would be better to isolate PT tissues and sequence them before and after the occurrence of PVTT to understand the molecular mechanisms involved in tumor progression.

## Conclusions

This study stratified patients according to the relative abundance of all cells within the tumor microenvironment. Five biologically meaningful subpopulations were separated, and the analysis mainly focused on comparing the molecular signature changes between the Pro-T group, the Mix group, and the Pro-Meta group. The prognosis of group Pro-T was the worst, and that of group Pro-Meta was the best. The results of WGCNA analysis and genes differential expression analysis exhibited high concordance. It was shown that primary HCCs already acquired an appropriate phenotype before they started the process of metastasis, reflected by a more suppressive immune microenvironment, more proliferation, and more active extracellular tissue activity of endothelial cells and myofibroblasts. HCC cells underwent further dedifferentiation in group Pro-Meta, which was specifically manifested by the downregulation of metabolic activities of various substances and disorders of the coagulation process, as well as the higher stemness characteristics. The increasing number of differential mutations implied that HCC cells have undergone stressful selection to adapt to the complex tumor microenvironment.

## Methods

### Clinical samples collection

The five native HCC tissue samples used in this study were obtained from patients undergoing surgery for HCC at the Affiliated Hospital of Guizhou Medical University (Guiyang, China). All participants in this study provided written informed consent. In concordance with the Declaration of Helsinki, this study was reviewed and approved by the ethics committee of the Affiliated Hospital of Guizhou Medical University (2,022,106). Frozen adjacent paracancerous tissues and PTs were obtained from five patients with HCC (*n* = 5, male, median age 55 years, HBV-positive, and no HCV infection detected). The majority of the PTs were larger than 5 cm (5 patients), and the Edmondson–Steiner histological grades were III and IV. For more detailed information, please refer to Table S1 (Additional file 1: Table S1). The clinical information of the single-cell dataset (GSE149614) used in this study is presented in Table S3 (Additional file 8: Table S3).

### Transcriptome analysis

A total amount of 1 µg RNA per sample was used as input material for the RNA sample preparations. Sequencing libraries were generated using the NEBNext® UltraTM RNA Library Prep Kit from NEB (New England Biolabs, USA) for Illumina®, following the manufacturer’s recommendations, and index codes were added to attribute sequences for each sample. Library preparations were sequenced on an Illumina Novaseq platform, and 150 bp paired-end reads were generated. First, FastQC (v0.11.9) [34] was used to evaluate the quality of raw data, and low-quality reads were filtered using TrimGalore (v0.6.7) [35]. Hisat2 (v2.2.1) [36] was used to map high-quality reads to the reference genome (hg19), and Htseq-count (v2.0.1) [37] was used to quantify the transcripts. Differentially expressed genes (DEGs) were identified using the DESeq2 (v1.32.0) [38] package. KEGG/GO enrichment analysis of the selected DEGs was conducted using the clusterProfiler (v4.05) package [39]. Because downstream analysis is entirely based on tumor samples, we did not perform data processing on adjacent tumor samples.

### Whole exome analysis

The exome sequences were efficiently enriched from 0.4 µg genomic DNA using an Agilent liquid capture system (Agilent SureSelect Human All Exon V6) according to the manufacturer’s protocol. The DNA library was sequenced on an Illumina system for paired-end 150 bp reads. Quality diagnostics of the sequencing data were performed using FastQC (v0.11.9) [34], and reads with adapter contamination were filtered using Trimmomatic (v0.39) [40]. Reads were aligned to the reference genome (UCSC.hg19.fasta) using BWA Mem (v0.7.17) [41]. Variant calling was performed according to the tutorial provided by Ulintz et al. [42], which was based on the Genome Analysis Toolkit (GATK, v4.2.6.1) platform [43]. For conservative calling of shared somatic mutations among multiple nodules, we removed the germline variant sites provided by gnomAD. The remaining variants were further annotated using the SnpEff (v5.1) software [44].

### Tumor microenvironment (TME) cell infiltration analysis

A single-cell RNA sequencing dataset was used to construct a signature matrix. The raw counts of gene expression data and related metadata were downloaded from the Gene Expression Omnibus (GEO) database with an accession number of GSE149614. The cell type information and cell markers used in this study were supplied in Table S4 (Additional file 9: Table S4), which completely came from the original article [9]. The total 53 cell clusters were separated into six main cell types, including hepatocyte, T/NK cell, myeloid cell, B cell, endothelial cell, and fibroblast according to classic cell markers (Additional file 10: Fig. S6). CIBERSORTx [45] was then used to estimate the abundance of known cell populations in bulk RNA-seq data from the TCGA-LIHC, LIRI-JP, and LICA-FR cohorts (primary tumor samples only). Raw counts from both the scRNA-Seq and bulk RNA-Seq data were used as the input for CIBERSORTx to ensure that both datasets were represented within the same normalization space. The ratios of different cell populations in the signature matrix were normalized to 100%. Statistically significant differences in the abundance of each cell type among the Pro-T, Mix, and Pro-Meta groups were determined using a two-tailed Student’s t-test. Statistical significance was set at *p* < 0.05. The TCGA-LIHC and LIRI-JP cohorts were used for Kaplan–Meier survival analysis.

### WGCNA

The batch effect between our and the TCGA-LIHC cohort RNA-Seq data was removed at the raw count level using the R package sva (v3.40.0) [46]. After normalizing the merged data from counts to transcripts per million (TPM), the data were transformed into log2(TPM + 1). For quality control, the top 5000 genes with higher median absolute deviation scores were retained for further analysis, and outlier samples were removed according to the clustering tree. The best soft threshold (power) was set to 8, which was used to construct the weighted correlation network using the blockwiseModules function in the R package WGCNA (v1.71) [47].

### Regulator activity analysis

The differential activities of transcription factors were analyzed using the Virtual Inference of Protein-activity by Enriched Regulon (VIPER) algorithm compiled in the R package viper (v1.26.0) [48]. First, the ARACNe-AP (v1.0.0) was used to generate a regulatory network for the LIHC cohort [49]. The transcription factors used in this study were obtained from https://github.com/califano-lab/PISCES/blob/master/data/human_regulators.rda. The regulatory network, along with the gene expression signature, was used as a model input for msVIPER. TPM quantification of Pro-T and Pro-Meta samples was used as the gene expression matrix for both ARACNe-AP and msVIPER.

### GSEA

GSEA was performed on 50 hallmark genesets, which were downloaded from the Molecular Signatures Database (MsigDB) [50]. ssGSEA was performed using the GenePattern platform to compare pathway activity among different groups [51]. The CELL_PROLIFERATION geneset was obtained from https://www.gsea-msigdb.org/gsea/msigdb/human/geneset/CELL_PROLIFERATION_GO_0008283. The ROESSLER_LIVER_CANCER_METASTASIS_UP geneset was obtained from https://www.gsea-msigdb.org/gsea/msigdb/human/geneset/ROESSLER_LIVER_CANCER_METASTASIS_UP. The HCC_CELL_STEMNESS geneset was obtained from 10.2147/SCCAA.S307043. The HCC_T_CELL_Exhausted_SCORE geneset was obtained from Yiming Lu et al. [9]. Liver-specific genesets were obtained from https://www.gsea-msigdb.org/gsea/msigdb/human/geneset/HSIAO_LIVER_SPECIFIC_GENES.

### Differential mutation analysis

The difference in gene mutations was calculated using Fisher’s exact test on a 2 × 2 contingency table generated from two independent groups using the function *mafCompare* in the R package maftools (v2.8.05) [52]. The null hypothesis was that the gene mutation frequency was independent of grouping. Statistical significance was set at *p* < 0.05.

### Domain enrichment analysis

Domain enrichment analysis was performed using the pfamDomains function in the R package maftools [52] to identify the most deregulated pathways and protein families involved in similar functions.

### Electronic supplementary material

Below is the link to the electronic supplementary material.

Supplementary Material 1

Supplementary Material 2

Supplementary Material 3

Supplementary Material 4

Supplementary Material 5

Supplementary Material 6

Supplementary Material 7

Supplementary Material 8

Supplementary Material 9

Supplementary Material 10

## Data Availability

The raw bulk RNA and whole exome sequencing (WES) data reported in this paper have been deposited in the Genome Sequence Archive in National Genomics Data Center, China National Center for Bioinformation / Beijing Institute of Genomics, Chinese Academy of Sciences (GSA-Human: HRA004490) that are publicly accessible at https://ngdc.cncb.ac.cn/gsa-human/s/uHpR1Mm5. For the TCGA-LIHC cohort, preprocessed bulk RNA-seq data and gene mutations table with MuTect2 annotation were downloaded from the GDC Data Portal (https://portal.gdc.cancer.gov). For LIRI-JP and LICA-FR cohorts, preprocessed bulk RNA-seq data were downloaded from the ICGC Data Portal (https://dcc.icgc.org). The single-cell RNA raw count and metadata are available from the Gene Expression Omnibus (GEO), the accession number is GSE149614. The code is available at GitHub at https://github.com/FangPrecisionlab/HCC_PVTT_PT.
