# Supplementary material for: Transcriptomic and genomic characteristics of intrahepatic metastases of primary liver cancer
Source: BMC Cancer. 2024 Jun 1;24:672. doi: 10.1186/s12885-024-12428-x (PMC11144329; doi:10.1186/s12885-024-12428-x)
Supplement: Supplementary file 2 — Supplementary Material 2 [file 12885_2024_12428_MOESM2_ESM.docx]

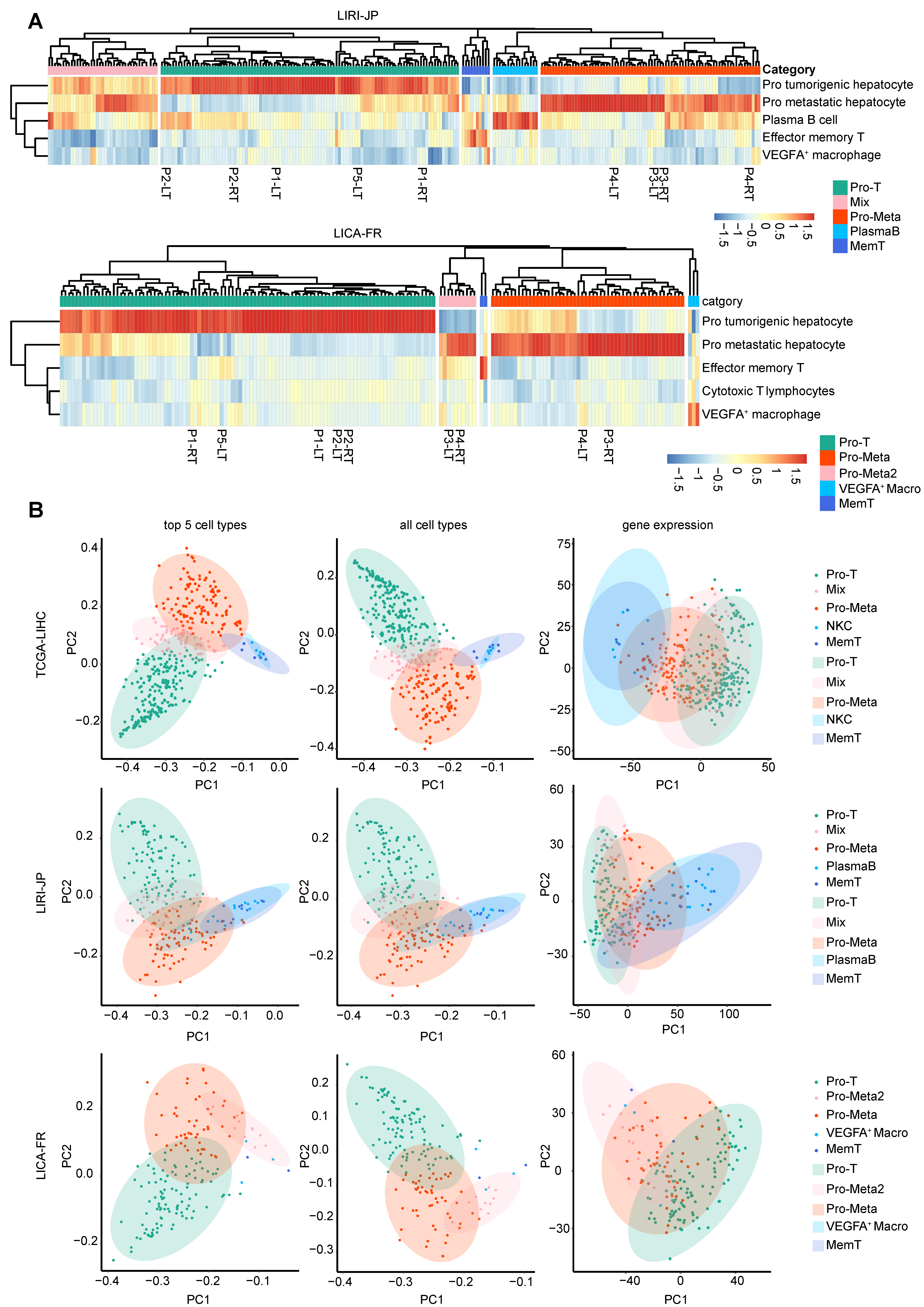


**Additional file 2: Fig. S1.** Stratification of patients with hepatocellular carcinoma (HCC) in three cohorts. **A** Hierarchical clustering of HCC samples based on top five relative cell abundances in cohort LIRI-JP (upper) and LICA-FR (lower). The native samples are highlighted. **B** PCA plot based on relative cell abundance of top five cell types (left), all cell types (middle), and gene expression of five HCC groups (right).
