# Supplementary material for: Transcriptomic and genomic characteristics of intrahepatic metastases of primary liver cancer
Source: BMC Cancer. 2024 Jun 1;24:672. doi: 10.1186/s12885-024-12428-x (PMC11144329; doi:10.1186/s12885-024-12428-x)
Supplement: Supplementary file 3 — Supplementary Material 3 [file 12885_2024_12428_MOESM3_ESM.docx]

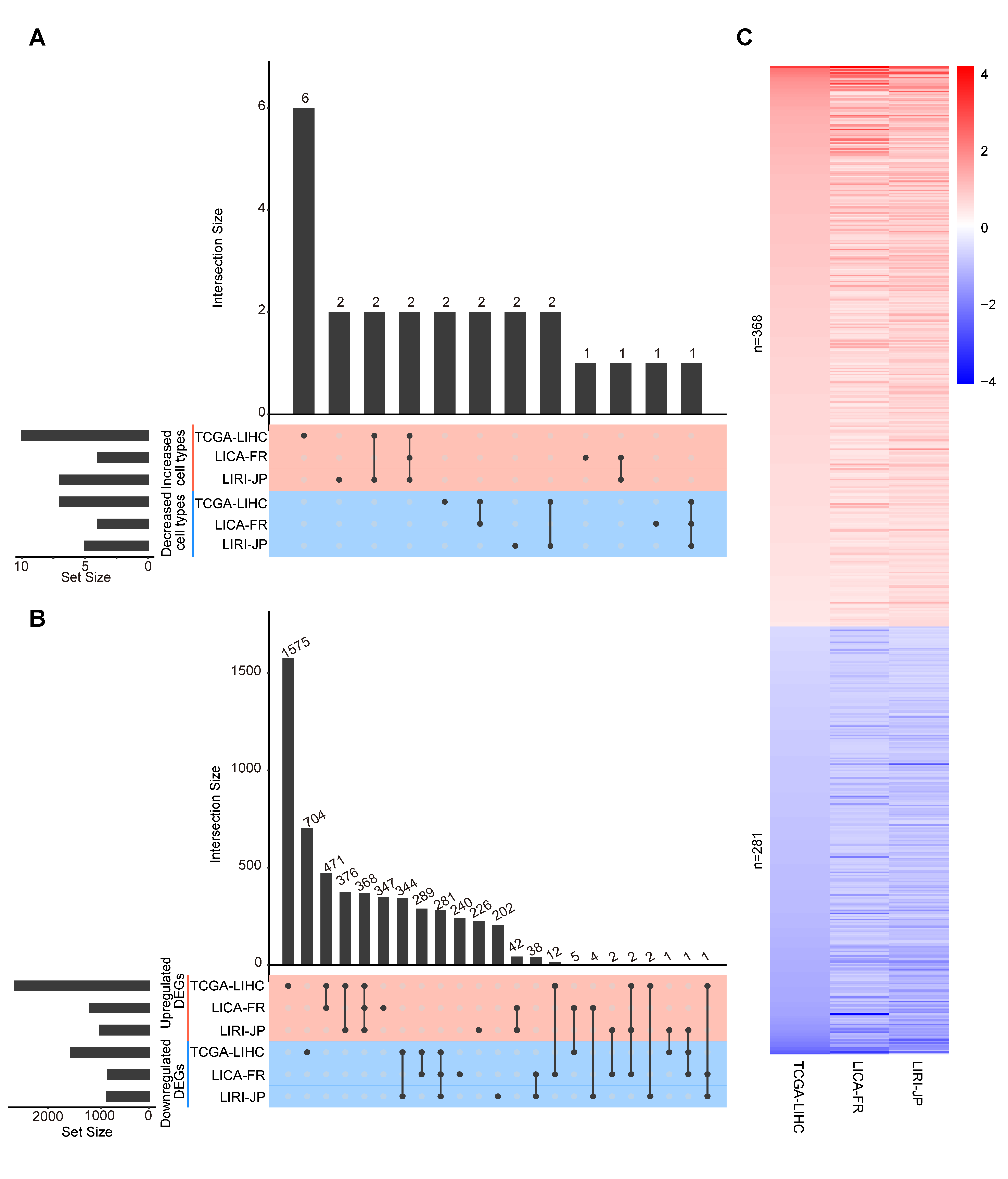


Additional file 2: Fig. S2. Comparison of cell components and differentially expressed genes (DEGs) among three cohorts. A The UpSet plot of increased or decreased cell components from group Pro-T to group Pro-Meta between three cohorts. B The UpSet plot of up- or down-regulated DEGs in group Pro-Meta between three cohorts. C The heatmap of log2FC of co-upregulated and co-downregulated DEGs in three cohorts.
