# Supplementary material for: Transcriptomic and genomic characteristics of intrahepatic metastases of primary liver cancer
Source: BMC Cancer. 2024 Jun 1;24:672. doi: 10.1186/s12885-024-12428-x (PMC11144329; doi:10.1186/s12885-024-12428-x)
Supplement: Supplementary file 4 — Supplementary Material 4 [file 12885_2024_12428_MOESM4_ESM.docx]

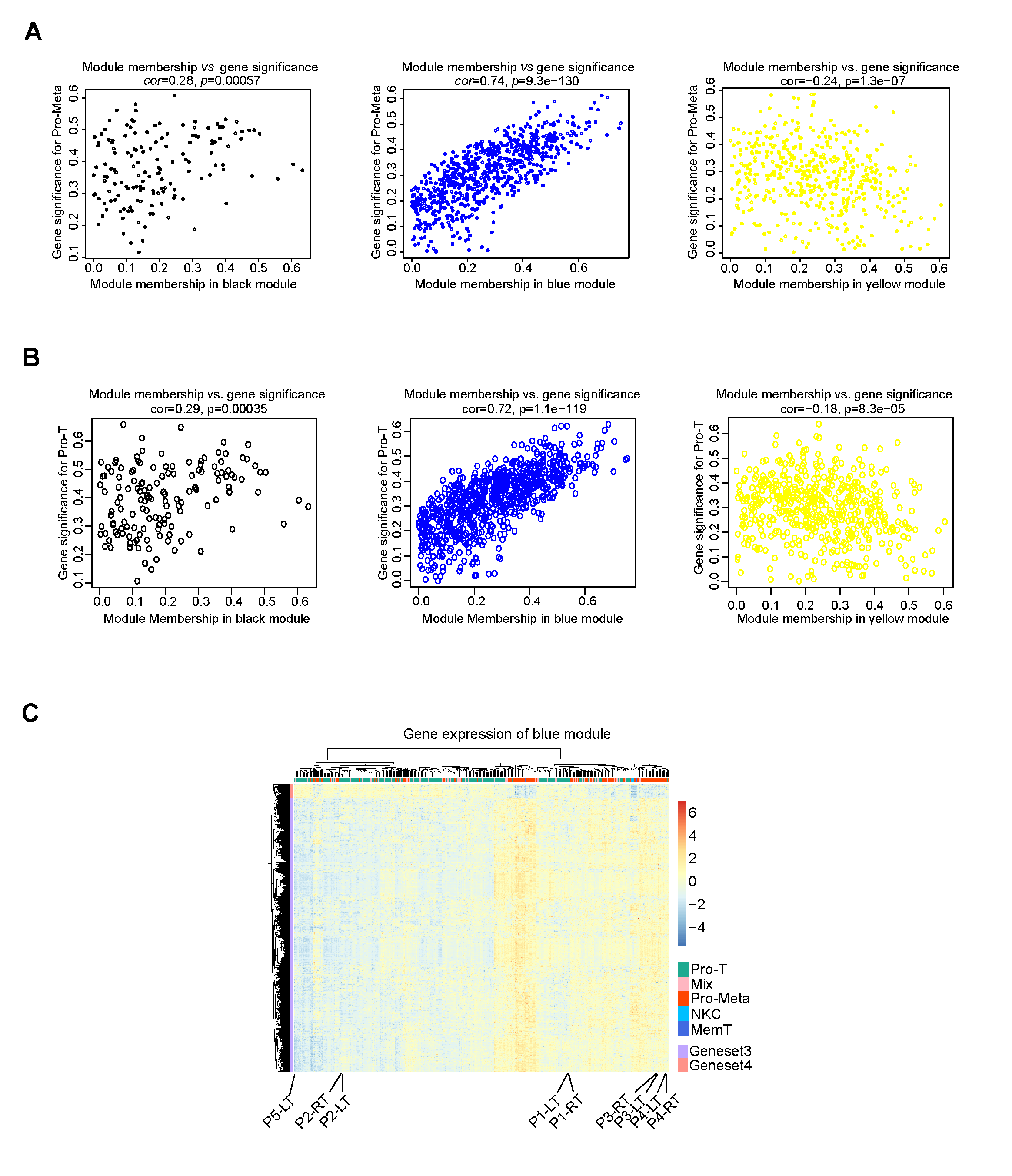


**Additional file 4: Fig. S3.** Weighted correlation network analysis (WGCNA) of the hepatocellular carcinoma (HCC) cohort. **A** Scatter chart for correlation analysis of module membership in black (left), blue (middle), and yellow (right) modules and gene significance for Pro-Meta group **(A)** and Pro-T group **(B)**. **C** Gene expression heatmap of genes in blue module.
