# Supplementary material for: Transcriptomic and genomic characteristics of intrahepatic metastases of primary liver cancer
Source: BMC Cancer. 2024 Jun 1;24:672. doi: 10.1186/s12885-024-12428-x (PMC11144329; doi:10.1186/s12885-024-12428-x)
Supplement: Supplementary file 6 — Supplementary Material 6 [file 12885_2024_12428_MOESM6_ESM.docx]

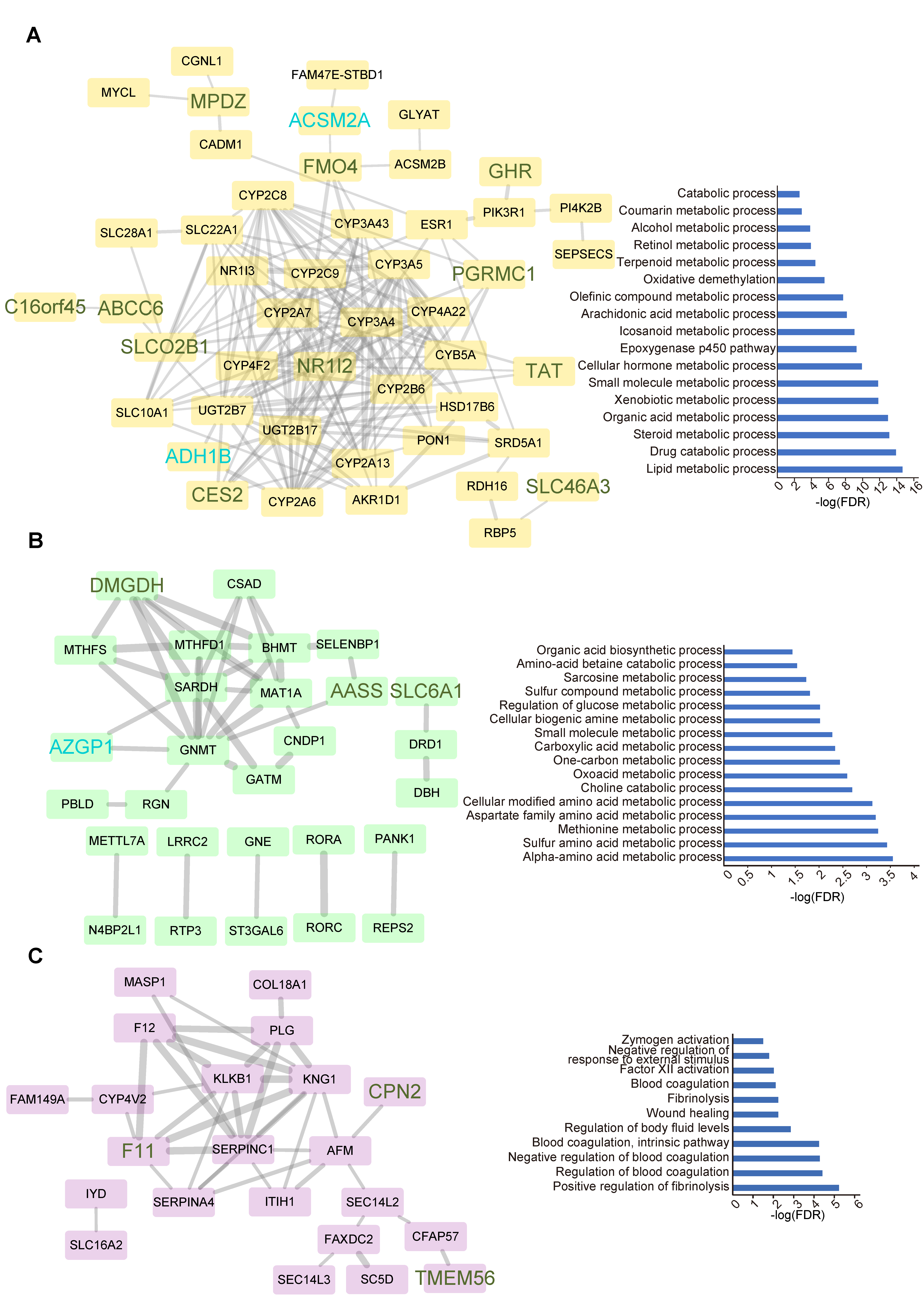


**Additional file 6: Fig. S5.** Protein-protein interaction (PPI) network analysis of gene modules upregulated in the Pro-T group. **A-C** PPI network analysis (left) and GO analysis (right) of three gene clusters (cluster T4-T6) upregulated in the Pro-T group identified by STRING clustering analysis. Genes in PPI network that intersected with the black and blue gene modules from WGCNA that are upregulated in the Pro-T group were colored in olive green and dark turquoise, respectively.
