# Supplementary material for: Transcriptomic and genomic characteristics of intrahepatic metastases of primary liver cancer
Source: BMC Cancer. 2024 Jun 1;24:672. doi: 10.1186/s12885-024-12428-x (PMC11144329; doi:10.1186/s12885-024-12428-x)
Supplement: Supplementary file 10 — Supplementary Material 10 [file 12885_2024_12428_MOESM10_ESM.docx]

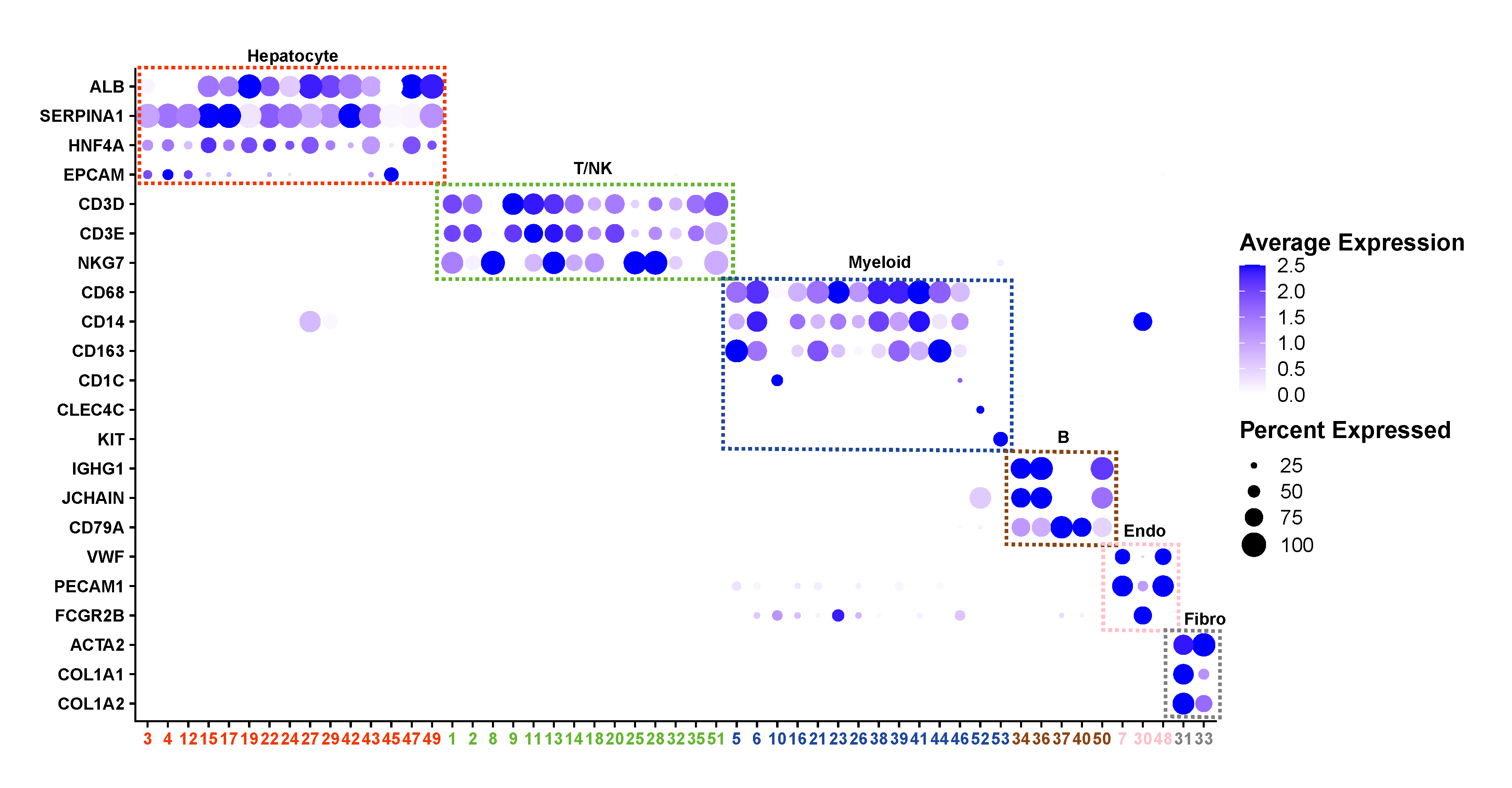


**Additional file 10: Fig. S6.** The average expression of classic markers and the percentage of expressed cells of eight cell subtypes.
